# Identification of intestinal parasites in wild American mink (*Neovison vison*) from Biebrza and Narew national parks (Poland)

**DOI:** 10.1007/s00436-023-07864-w

**Published:** 2023-05-16

**Authors:** Maciej Klockiewicz, Tadeusz Jakubowski, Justyna Karabowicz, Piotr Bąska, Justyna Winiarska, Ewa Długosz

**Affiliations:** 1 Department of Pre-clinical Sciences, Institute of Veterinary Medicine, Warsaw University of Life Sciences, Ciszewskiego St. 8, 02-786 Warsaw, Poland; 2 Laboratory of the Polish Society of Breeders and Producers of Fur Animals, Pocztowa St. 5, 62-080 Tarnowo Podgórne, Poland

**Keywords:** Wild American mink (*Neovison vison*), Gastrointestinal parasites, Prevalence, Intensity, A reservoir of infection

## Abstract

American mink (*Neovison vison*) is an invasive species in the sylvatic environment of Poland. Mink are exposed to different parasite infections as their preys serve as intermediate and/or paratenic hosts. The study aimed to discriminate the pattern of intestinal parasite infections in mink inhabiting Biebrza (BNP) and Narew (NNP) national parks. Gastrointestinal tract examinations revealed Coccidia, Echinostomatidae, Taenidae, and Capillariidae parasites. There was no significant difference in the parasite burden of mink, but patterns of infections varied between both localizations. Coccidia were found in 3.8% of BNP vs. 6.7% of NNP mink. Fluke prevalence was significantly higher in NNP 27.5% compared to 7.7% in BNP mink. Tapeworms were only found in 3.4% of NNP mink. Significantly more *Aonchotheca* eggs were found in BNP 34.6% vs. 11.4% in NNP mink. The intensity of coccidiosis and aonchothecosis was low in both parks. Fluke intensity varied between low to moderate (ranging from 1 to 16) in BNP and low to massive (ranging from 1 to 117) in NNP mink. Coinfections of various parasite species were noted in both areas. Morphological and DNA analysis revealed that flukes belonged to *Isthiomorpha melis* and tapeworms to *Versteria mustelae*. It was the first isolation of *V. mustelae* in mink of those localizations. In conclusion, our study showed that mink indwelling Biebrza and Narew national parks are moderately infested with parasites. Results suggest that mink play an important role as a reservoir for parasites endangering endemic mustelids, becoming also a potential risk factor in case of accidental transmissions to farm mink. That is why, more strict biosecurity measures are required to protect farm mink.

## Introduction

The wild American mink (*N. vison*) is a widespread mustelid predator in Poland’s feral and forest environment. Skorupski (2016) published his critical analysis of the nature conservation and invasion aspects of the mink. Reporting earlier studies, the author cited that the first animals living in the wild were observed in 1954 (Ruprecht et al. 1983), and later at the beginning of the 1960s (Brzeziński and Marzec 2003). Next, hunting reports confirmed the presence of mink in at least 34% and 50% of hunting circuits in Poland, respectively (Kamieniarz and Panek 2008; Grabońska 2011). Mink occur almost throughout the country, except in the southeastern part (Brzeziński et al. 2019). It is accepted that the mink population established in Poland originates from two main sources. Some arrived from the former USSR republics (e.g., Belarus and Lithuania), where they had been previously introduced for rearing and/or hunting. Others were descendants of those, which had accidentally escaped from the local farm facilities and survived in the wild. Despite the origin, American mink are also present in many localizations across Europe (Bonesia and Palazon 2007). Large populations of non-native species in the natural environment significantly affect the number of native animal species, thus reducing the biodiversity of ecosystems, especially protected ones, such as national parks, nature reserves, and Natura 2000 areas (Vilà and Hulme 2017).

Mink are carnivorous predators which live in semi-aquatic and/or terrestrial environments. They hunt on prey—a wide range of victims such as freshwater fish, amphibians (mostly frogs), birds, and small mammals (rodents, shrews, etc.). Their menu includes various invertebrates such as slugs and snails, crayfish, and big insects (Jędrzejewska and Jędrzejewski 2001). It was observed that mink strongly influenced the balance between predators and prey (Carlsson et al. 2010; McDonald et al. 2007). For example, their expansion is correlated with serious declines of several water birds and semi-aquatic mammals (Brzeziński et al. 2010, 2012).

Mink living in the wild are naturally exposed to several causative agents responsible for infectious and parasitic diseases. Among these, parasites such as protozoans, trematodes, cestodes, nematodes, and acanthocephalans are likely to infect the mink. Thus, wild mink are a reservoir of parasitic infections, which might be transmitted to farmed animals via vectors such as birds or small rodents. Moreover, these infections would seriously affect farm mink health and welfare conditions.

The study aimed to discriminate the set of parasite infections that are currently occurring in wild American mink (*N. vison*) inhabiting two national parks in the northeast of Poland. As the mink have definitively a bad impact on the natural resources of the endemic fauna competing with indigenous predators, being also responsible for some damage within native populations of prey such as birds, fish, and small mammals. Mink are monitored and selectively trapped to control the population limit.

## Material and methods

### Animals

Wild American mink (*N. vison*) were obtained from Biebrza and Narew national parks by the year 2018–2020. Mink trapping was a continuation part of conservation measures according to the project LIFE + 09/NAT/PL/000263 realized by the national parks. Mink were caught alive following ethical trapping standards. The collection of animals was done twice a year in spring and autumn. Animals were subsequently euthanized with CO_2_, and bodies were stored frozen at – 20 °C. Overall, 175 mink: 26 animals from BNP (4 in 2018; 13 in 2019; and 9 in 2020) and 149 animals from NNP (79 in 2018; 52 in 2019; and 18 in 2020) were investigated, and post-mortem examinations were performed.

### Detection of endoparasites

Parasitological examinations were conducted on the isolated gastrointestinal tracts. The stomach and small and large intestines content was investigated macroscopically followed by the standard flotation method with supersaturated NaCl solution. Dispersive objects such as eggs, oocysts, and intestinal worms were observed and recorded by the Opta-Tech diagnostics system: light microscope MD-200 and stereomicroscope SK equipped with HDMI cameras.

### Statistical analysis

Negative mink from both parks were classified as non-infected, whereas animals harbouring at least one parasite species were classified as infected ones. To compare the prevalence of infections between both parks, the Parson’s χ^2^ test was used. The prevalences of mink infected with particular parasite species (coccidia, flukes. or nematodes) between both Parks were also analyzed using Parson’s χ^2^ test.

### Molecular identification of fluke and tapeworm species

Isolated specimens of flukes and tapeworms were washed with 0.9% NaCl and stored at – 20 °C until examined. Total genomic DNA was extracted from frozen tissue using a Genomic Mini DNA isolation kit (AA Biotechnology, Poland) according to the manufacturer’s instructions. The DNA was eluted with 50 μl of elution buffer, following the use of 10 μl of the eluate as a template for the PCR reaction. Gene primers specific to the mitochondrial cytochrome oxidase I (COI) were used in the study: F: 5′TTTTTTGGGCATCCTGAGGTTTAT3′ and R: 5′TAAAGAAAGAACATAATGAAAATG3′ (Bowles et al. 1995).

PCR products were amplified using Color OptiTaq PCR Master Mix (Eurx, Poland) under the following conditions: initial denaturation at 95 °C for 5 min followed by 40 cycles of denaturation at 95 °C for 30 s and annealing at 55 °C for 60 s and 72 °C for 60 s, followed by a post-amplification extension for 5 min at 72 °C. The PCR products were resolved in 1% agarose gel and excised and purified using GeneElute gel extraction kit (Sigma-Aldrich) according to the manufacturer’s instructions. The purified DNA fragments were sequenced by a commercial service (Genomed) and analyzed by Basic Local Alignment Search Tool (BLAST) using sequences from the National Center for Biotechnology Information database (https://blast.ncbi.nlm.nih.gov/Blast.cgi). Sequences showing the highest similarity were aligned with fluke and tapeworm COI nucleotide fragments using CLUSTALW (https://www.genome.jp/tools-bin/clustalw). Alignment and phylogenetic reconstructions were performed using the function “build” of ETE3 v3.1.1 (Huerta-Cepas et al. 2016) as implemented on the GenomeNet (https://www.genome.jp/tools/ete/). Phylogenetic trees were constructed using maximum likelihood (ML) algorithms PhyML v20160115. Branch supports were computed out of 100 bootstrapped trees.

## Results

### Parasitological examination of wild American mink from BNP

A total number of 26 mink from the BNP were analyzed. Examination of gastrointestinal tracts from 4 animals collected in 2018 revealed the presence of only a single fluke in a female mink and *Aonchotheca* sp. eggs in another female mink. In 2019, 13 mink were examined, and the *Aonchotheca* sp. eggs were only found in 2 female and 5 male mink. In 2020, *Aonchotheca* sp. eggs and coccidia oocysts were only found in 1 male mink. The same specimen harboured 16 intestinal flukes.

The intensity of aonchothecosis and coccidiosis in particular hosts was estimated at a low level, mostly single oocysts of *Coccidia* or *Aonchotheca*-like eggs were found. The intensity and prevalence of fluke infection were estimated as low as the trematodes were only found in 2 mink. Data are summarized in Table 1.

**Table 1:** Occurrence of gastrointestinal parasite infections in wild mink from Biebrza National Park

	Protozoans/Coccidia			Trematodes			Cestodes			Nematodes		
	♀	♂	Σ	♀	♂	Σ	♀	♂	'Σ	♀	♂	Σ
2018 4 mink	0/4	0/0	0/4	1/4	0/4	1/4	0/4	0/0	0/4	1/4	0/0	1/4
2019 13 mink	0/3	0/10	0/13	0/3	0/10	0/13	0/3	0/10	0/13	2/3	5/10	7/13
2020 9 mink	1/4	0/5	1/9	0/4	1/5	1/9	0/4	0/5	0/9	0/4	1/5	1/9
Prevalence/sex	9%	0		9%	0		0	0		27.3%	40%	
Total number: 26 mink	Total prevalence:		3.8%	Total prevalence:		7.7%	Total prevalence:		0	Total prevalence:		34.6%

### Parasitological examination of wild American mink from NNP

A total number of 149 mink from NNP were investigated. Examination of gastrointestinal tracts from 79 animals collected in 2018 revealed the presence of coccidian oocysts in 1 female and 5 male mink, intestinal flukes in 1 female and 6 male mink, and *Aonchotheca* sp. eggs in 6 female mink. Coinfections of coccidia and *Aonchotheca* sp. or coccidia and flukes were noted in single female and male mink, respectively. Next, 52 mink were examined in 2019. Coccidian oocysts were only found in 2 male mink, intestinal flukes in 6 female and 15 male mink, and a single tapeworm in 1 female and 1 male mink. The *Aonchotheca* eggs were observed in 1 female and 5 male mink. The coinfection of flukes and tapeworms occurred in 1 female and 1 male mink. Another single male mink was found infected with flukes and *Aonchotheca* sp. eggs. Mixed infection of coccidia, flukes, and *Aonchotheca* sp. was noted only in 1 male individual. In 2020, coccidian oocysts were found in 2 male mink only, intestinal flukes in 4 female and 9 male mink, tapeworms in 1 female and 2 male mink, and *Aonchotheca* sp. eggs in 4 male mink. A mixed infection of coccidia and intestinal flukes was found in 1 male mink. A coinfection of *Aonchotheca* sp. and flukes was found in 3 male mink. A single female and male mink were found coinfected with flukes and tapeworms. The coinfection of coccidia, flukes, tapeworms, and *Aonchotheca* nematodes occurred only in another 1 male host.

The level of intestinal coccidiosis and aonchothecosis was estimated as low. There were few coccidian oocysts or *Aonchotheca*-like eggs usually found by the flotation method in particular hosts. The intensity of fluke infection varied from 1 to 117 specimens per host; therefore, the intensity was estimated as low to massive. Altogether, 883 flukes were isolated and the average flukeworm burden was 20.3 specimens per host. The average intensity in female mink was 16.2 flukes (from 1 to 67) and 23.5 flukes in the male host (from 1 to 117). The number of tapeworms varied from 1 to 3 per host. Summarized data concerning particular infections are presented in Table 2.

**Table 2:** Occurrence of gastrointestinal parasite infections in wild mink from Narew National Park

	Protozoans			Trematodes			Cestodes			Nematodes		
	♀	♂	Σ	♀	♂	Σ	♀	♂	Σ	♀	♂	Σ
2018 79 mink	1/39	5/40	6/79	1/39	6/40	7/79	0/39	0/40	0/79	6/39	0/40	6/79
2019 52 mink	0/18	2/34	2/52	6/18	15/34	21/52	1/18	1/34	2/52	1/18	6/34	7/52
2020 18 mink	0/7	2/11	2/18	4/7	9/11	13/18	1/7	2/11	3/18	0/7	4/11	4/18
Prevalence/sex	1.6%	10.6%		17.2%	35.3%		3.1%	3,5%		10.9%	11.8%	-
Total number: 149 mink	Total prevalence:		6.7%	Total prevalence:		27.5%	Total prevalence:		3.4%	Total prevalence:		11.4%

### Statistical analysis of parasite prevalence

The prevalence of infections with at least one parasite species did not significantly differ between the two groups of examined mink and was calculated as 42.3% and 36.9% in animals inhabiting BNP and NNP, respectively. Similarly, the prevalence of coccidia (3.9% in BNP and 6.7% in NNP) was not significantly different. However, a significantly higher number of mink was infected with flukes (*p* < 0.05) NNP 27.5% compared to BNP 7.7%. On contrary, mink from NNP were less frequently infected with *Aonchotheca* sp. 11.4% than BNP mink BNP 34.6%. The results are shown in Table 3.

**Table 3:** Prevalence of mink infected with parasites in BNP and NNP—animals infected with any parasite were described as “infected” and analyzed

Parasite	Prevalence [%]	
	Biebrza National Park	Narew National Park
Infected	42.3	36.7
Protozoa (coccidia)	3.8	6.7
Trematodes	7.7^*^	27.5
Cestodes	0	3.4
Nematodes	34.6^*^	11.4

^*^Statistically significance (*p* < 0.05)

### Morphological investigation of trematodes and cestodes

#### Trematodes

Ten specimens were randomly chosen from all collected flukes to measure the body size. The average length of 5.26 mm (range: 3.94 to 6.16) and width of 0.85 mm (range: 0.56 to 1.12) were noted. Characteristic morphological features of the fluke’s body structures such as arrangements of both acetabula (oral and abdominal), testes shape, and position regarding the uterus and many hooks surrounding the anterior part of the body were analyzed. Parasites were pre-classified as *Isthmiophora melis* (a digenean fluke, Echinostomatidae family).

#### Cestodes

The body condition of 3 specimens of tapeworms recovered was good enough to measure the length of the strobila. The average strobila length was 25.9 cm (from 23.6 to 27.3 cm). Regrettably, scolexes of tapeworms were found affected by long-term freezing and thawing. The structure of the scolex, size of strobila and shape, and internal composition of proglottids resembled *Versteria mustelae* tapeworm.

### Molecular identification

To confirm the species of the collected parasites, molecular analysis of mitochondrial DNA was performed. A total number of 21 amplified fluke COI fragments (410 bp long) and 4 tapeworm COI fragments (392 bp long) were sequenced followed by deposition in GenBank under accession numbers MW476492-MW476516.

**Fig. 1:**
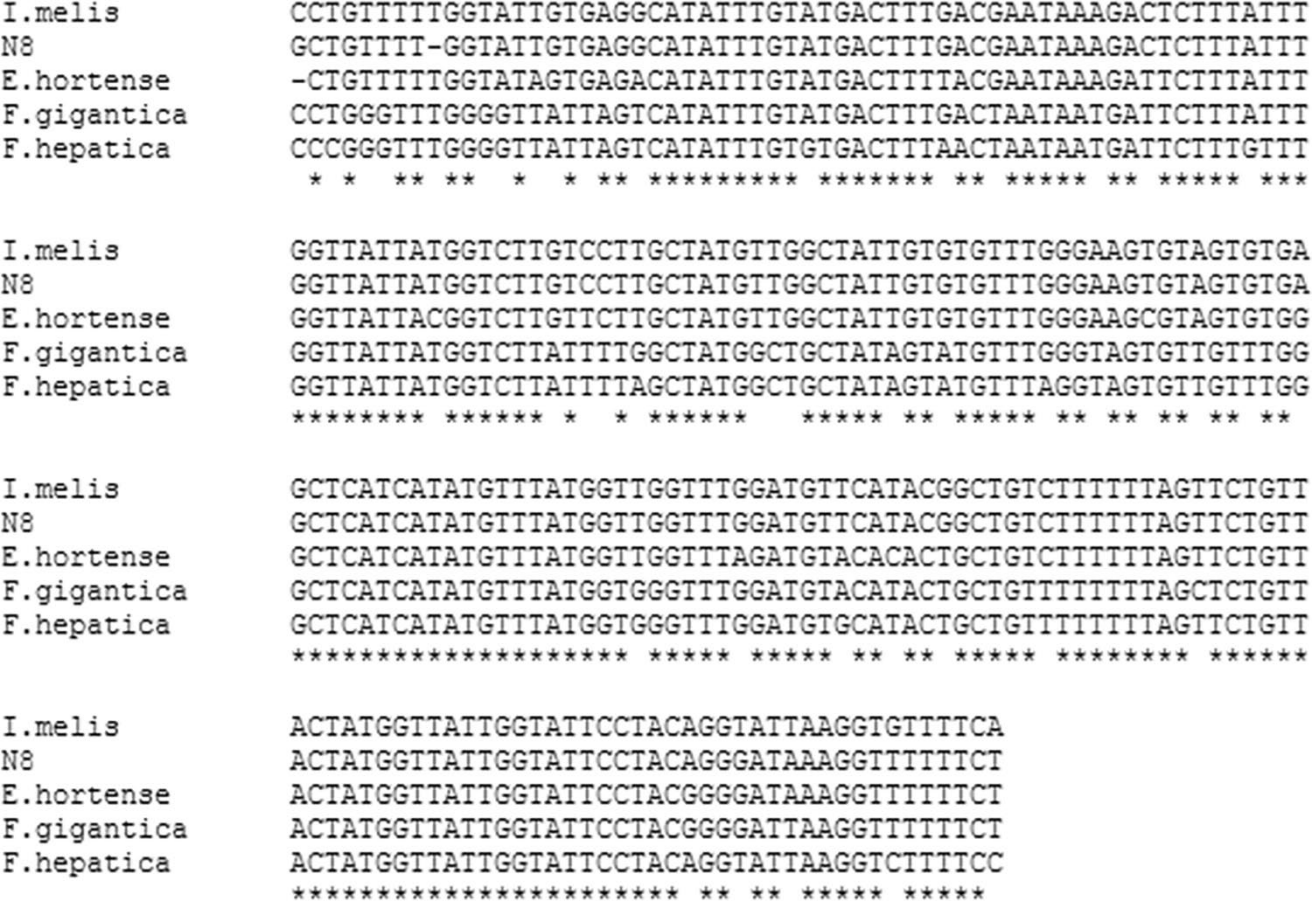
CLUSTALW Multiple alignment of N8 fluke isolate COI sequence (GenBank no. MW476492) with corresponding sequences from *Isthmiophora melis* (GenBank no. KT359581), *Echinostoma hortense* (GenBank no. KR062182), *Fasciola gigantica* (GenBank no. KT347282), and *Fasciola hepatica* (GenBank no. MN507460)

**Fig. 2:**
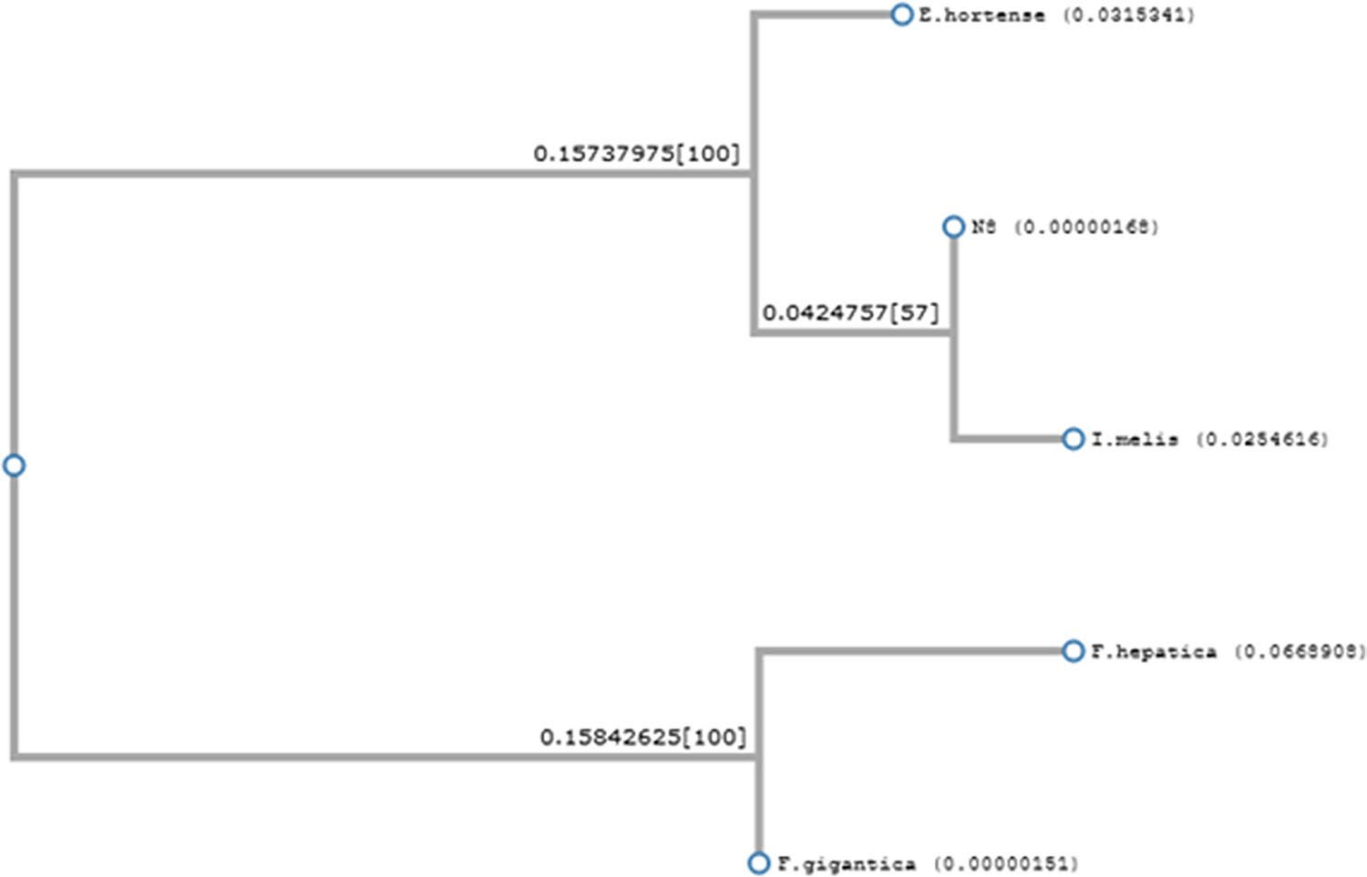
Phylogenetic analysis of N8 fluke isolate COI sequence (GenBank no. MW476492) with corresponding sequences from *Isthmiophora melis* (GenBank no. KT359581), *Echinostoma hortense* (GenBank no. KR062182), *Fasciola gigantica* (GenBank no. KT347282), and *F. hepatica* (GenBank no. MN507460)

DNA sequences originating from flukes were analyzed by BLAST and showed the closest similarity to COI sequences of *Isthmiophora melis* (GenBank no. KT359581) (95.98–98.18% identity), *Echinostoma hortense* (GenBank no. KR062182) (88.48–90.98% identity), *Fasciola gigantica* (GenBank no. KT347282) (83.5–84.48% identity), and *F. hepatica* (GenBank no. MN507460) (82.96–83.955% identity). Multiple alignments of these sequences are shown in Fig. 1. Because the *I. melis* sequence available in GenBank was about 190 bp shorter than our amplified sequences, all sequences were trimmed to the length of the shortest sequence before the construction of multiple alignments and phylogenetic trees. The phylogenetic analysis showed that all fluke sequences analyzed in the study are the most homological to *I. melis*. One representative phylogenetic tree is shown in Fig. 2.

**Fig. 3:**
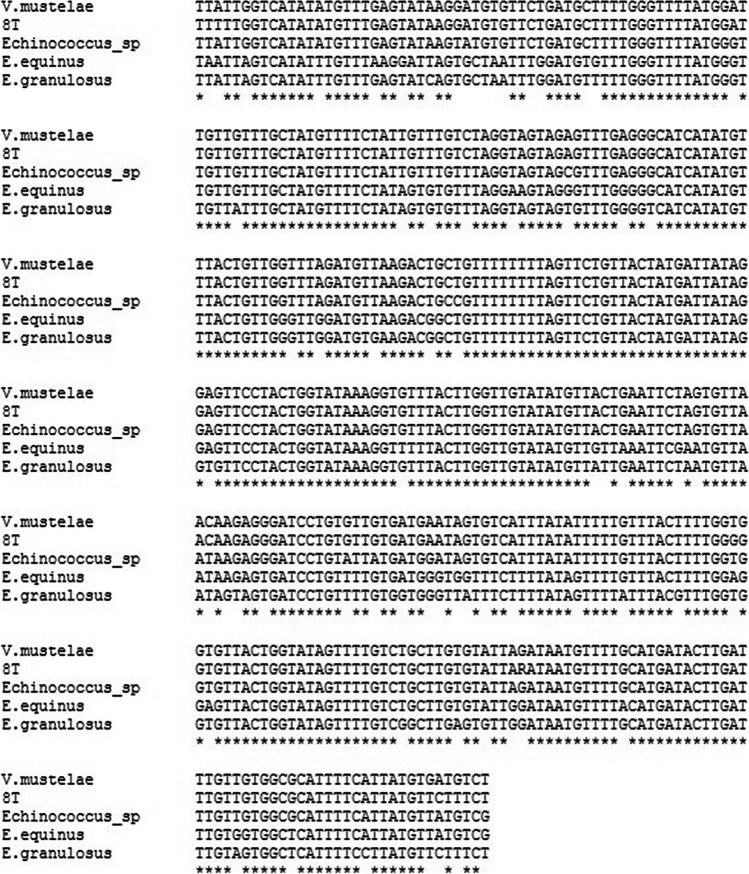
CLUSTALW multiple alignment of 8 T tapeworm isolate COI sequence (MW476515) with corresponding sequences from *Versteria mustelae* (GenBank no. AB732957), *Echinococcus* sp. (GenBank no. JQ690286), *E. equinus* (GenBank no. MN787562), and *E. granulosus* (GenBank no. MH428014)

**Fig. 4:**
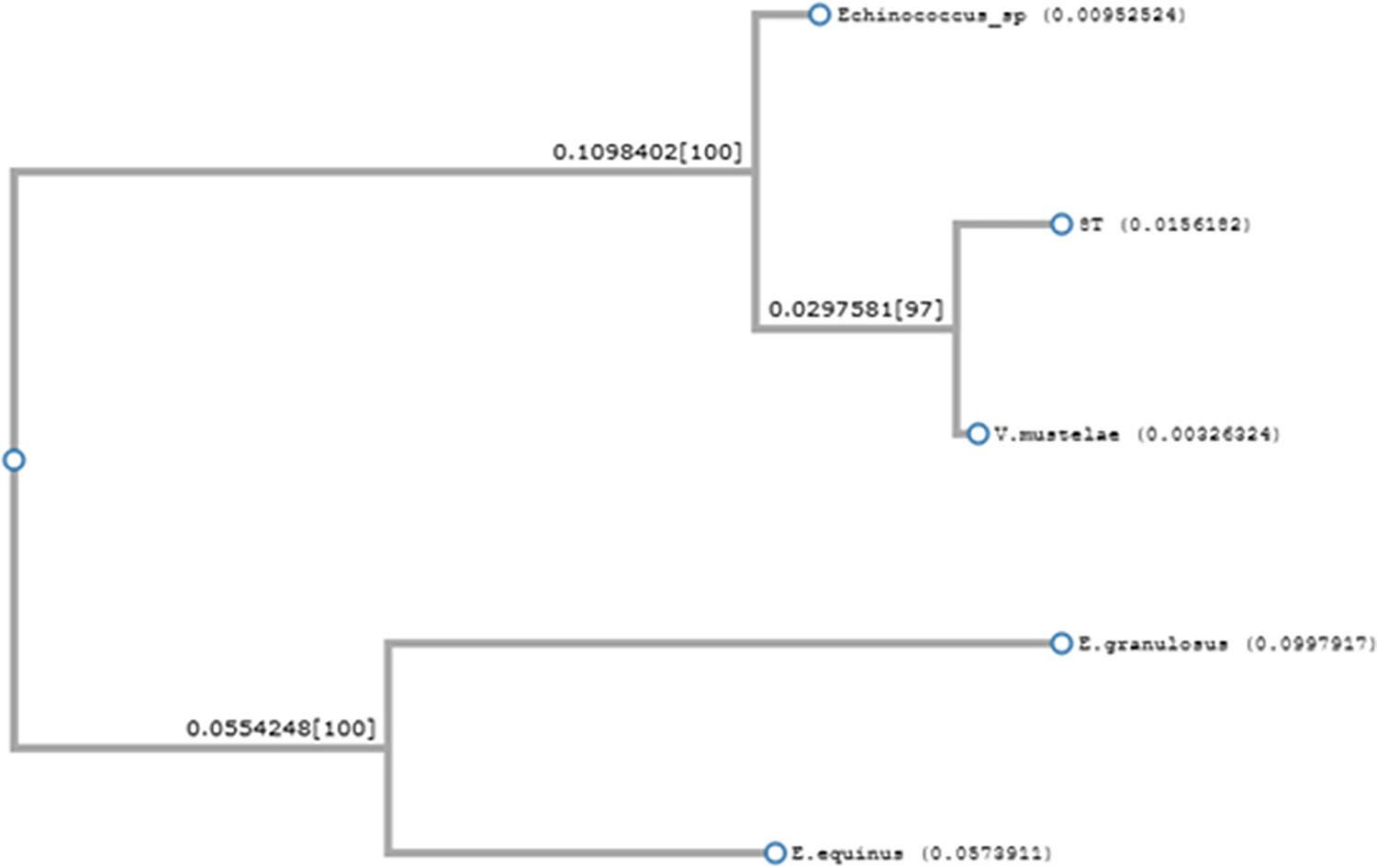
Phylogenetic analysis of 8 T tapeworm isolate COI sequence (GenBank no. 476515) with corresponding sequences from *Versteria mustelae* (GenBank no. AB732957), *Echinococcus* sp. (GenBank no. JQ690286), *E. equinus* (GenBank no. MN787562), and *E. granulosus* (GenBank no. MH428014)

DNA fragments amplified from tapeworms showed the highest similarity to COI sequences of *Versteria mustelae* (GenBank no. AB732957) (91.64–99.74% identity), *Echinococcus* sp. (GenBank no. JQ690286) (96.89–97.41% identity), *E. equinus* (GenBank no. MN787562) (87.82–90.23% identity), and *E. granulosus* (GenBank no. MH428014) (87.02–87.82% identity). Multiple alignments of these sequences are shown in Fig. 3. The phylogenetic reconstruction confirmed that analyzed tapeworm fragments are most homological to *V. mustelae* COI sequence. One representative phylogenetic tree is shown in Fig. 4.

## Discussion

A 3-year study revealed different patterns of the gastrointestinal parasite fauna in wild American mink (*N. vison*) populations living in NNP compared to those in BNP. Mink being active predators are exposed to a wide variety of autochthonous parasites species and may act as hosts. GI tract examination showed more diverse and numerous parasite populations in NNP minks than in BNP minks, ranging from coccidia, trematodes, and cestodes to nematodes.

The study results showed that out of protozoans, only coccidia oocysts were found in wild mink and occurred in 3.8% and 6.7% of mink living in BNP and NNP, respectively. The infection occurred in both parks, but it was not possible to identify the genera or species of coccidia involved. Several coccidia species were reported in farm mink, e.g., *E. furonis* and *E. hiepei* (Duszynski et al. 2000); *E. mustelae* (Duszynski et al. 2000; Gorham 2005; Kowalska 2015); *E. vison* (Duszynski et al. 2000); and *E. vison-*like (Kowalska 2015; Petersen et al. 2018). But coccidia of the genera *Isospora*/*Cystoisospora* were identified as *I. bigemina* (Gorham 2005) or *I. laidawi* (Gorham 2005; Kowalska 2015).

*Isthmiophora melis* flukes were the most prevalent parasite in the NNP mink population. The life cycle of these flukes in an aquatic environment involves the first intermediate host, the Lymnaeid water snail, and the second one, a freshwater fish or tadpole. Mink by consuming metacercariae present in the tissues of the second intermediate host becomes the definitive host. Higher numbers of trematodes found in gastrointestinal tracts of NNP mink might be associated with a larger fish population in the Narew river. It could be supported by our post-mortem findings—more fragments of undigested fish bodies (bones and scales) were found in the guts of NNP mink than in BNP ones (however statistically insignificant). Moreover, a higher abundance of fluke infection in those mink could be also explained by the fact that individuals had different eating habits and the mink traps in NNP were mostly placed on floating rafts.

Although the natural environment is similar in both national parks, a larger variety of parasites was found in the NNP mink, which presented in larger numbers of intestinal flukes and tapeworms than BNP mink. There are many potential intermediate hosts of *I. melis* flukes living in both localisations, but the animal-host species composition seems to be slightly different. Obtained results revealed that the prevalence of flukes in BBP mink was almost four times lower than in NNP mink (7.7% vs. 27.5%). Accordingly, animals from NNP harboured more flukes. This indicates that the NNP mink diet was mostly based on fish and amphibians.

Morphological and DNA analyses revealed that all the isolated flukes belonged to *Isthmiophora melis* species. Some trematode eggs were previously isolated in 37.5% of examined mink from Białowieża Primeval Forest, but the species was not determined (Górski et al. 2006). The *I. melis* fluke was also recognized as a dominating parasite species among helminths in wild American mink in Lithuania (Naugaraitė et al. 2014). Continuing their studies, the authors observed greater parasite species diversity in mink from wetlands in comparison to the forest ones and stated that those helminths were typical for the local mustelid hosts (Naugaraitė et al. 2019). The *I. melis* flukes obtained from different hosts (badger, American mink, hedgehog, and striped filed mouse) were intensively investigated by Hildebrand et al. (2015), and the study concluded that parasite specimens may show some morphological traits depending on their hosts. The intestinal flukes may cause gastroenteritis in affected animals. Thus, some moderately expressed gut lesions were noted in examined mink.

Single tapeworms were isolated only in NNP mink. Based on morphology and DNA analysis, parasites were identified as *Vesteria mustelae* species*.* Fact that only NNP mink harboured tapeworms is unlikely to be explained by the environmental conditions, but rather due to the feeding habits of the investigated individuals. The parasite life cycle involves a set of intermediate hosts, mostly small rodents, which are present in both areas. The *V. mustelae* was reported in various Mustelidae species, e.g., otter, ermine, long-tailed weasels, but its larval form, cysticercus, was even identified as an etiologic agent of the fatal course of infection in zoo monkeys in the USA (Lee et al. 2016). The larval stage was also noted to be involved in wildlife-transmitted *cysticercosis* and *coenurosis* in human beings and other primates (Deplazes et al. 2019). To our best knowledge, this tapeworm has not yet been isolated nor identified by DNA methods in wild American mink or other closely related mustelids in both investigated regions.

There were only single *Aonchotheca*-like eggs found by the flotation method in particular individuals: 34.6% in BNP and 11.4% in NNP mink, respectively. Unfortunately, there were no adult worms isolated from the guts. Thus, the assumption was made that eggs were produced by *Aonchotheca putorii*, formerly named *Capillaria putorii*. The *A. putorii* parasitizes in the stomach and intestines of mustelids and other wild carnivorans. It was reported in Canada in hosts closely related to the mink, such as short-tailed weasel (*Mustela erminea*), fisher (*Pekania pennanti*), marten (*Martes americana*), striped skunk (*Mephitis mephitis*), raccoon (*Procyon lotor*), and American mink itself (Butterworth and Beverley-Burton 1980). Zabiega (1996) found those parasites in over 1/3 of examined mink in the Southern part of Illinois State, USA. The *A. putorii* was also found in the French population of European mink (*Mustela lutreola*), European polecat (*Mustela putorius*), and in introduced wild American mink (Torres et al. 2008). There was a 54% prevalence of *A. putorii* reported in wild mink in the Galicia region of Spain (Martinez-Rondan et al. 2017). In the vicinity of the studied national parks, the *A. putorii* eggs (named *Capillaria* spp.) were preliminarily reported in wild American mink from Białowieża Forest in Poland (Górski and Łakomy 2004) and later confirmed in 31.3% of individuals (Górski et al. 2006). A bit north, the parasite was noticed in wild mink in Lithuania (Naugaraitė et al. 2014). More recently, *A. putorii* was described as a dominating gastrointestinal nematode species in non-native wild American mink, which had been examined from 2005 to 2017 in Poland (Kołodziej-Sobocińska et al. 2021). However, it should be also taken into consideration that eggs of similar shape may be found in a host infected with *Eucoleus aerophila* (previously *Capillaria aerophila*)*.* The details of its morphology were described by Butterworth and Beverley-Burton (1980), who characterized that nematode as parasitizing on tracheal and bronchial mucosa in red fox (*Vulpes vulpes*) and marten (*Martes* sp.).

## Conclusion

Our observations suggest that wild American mink plays an important role as a natural reservoir for various parasite pathogens, endangering other endemic mustelid species, also becoming a potential risk factor for accidental transmissions to farm mink. This highlights the need for stringent biosecurity measures to protect farmed mink.

## Data Availability

The material obtained in this study is stored at the Institute of Veterinary Medicine, Warsaw University of Life Sciences. Representative nucleotide sequences obtained in this study were submitted to GeneBank® under the accession numbers MW476492-MW476516.
